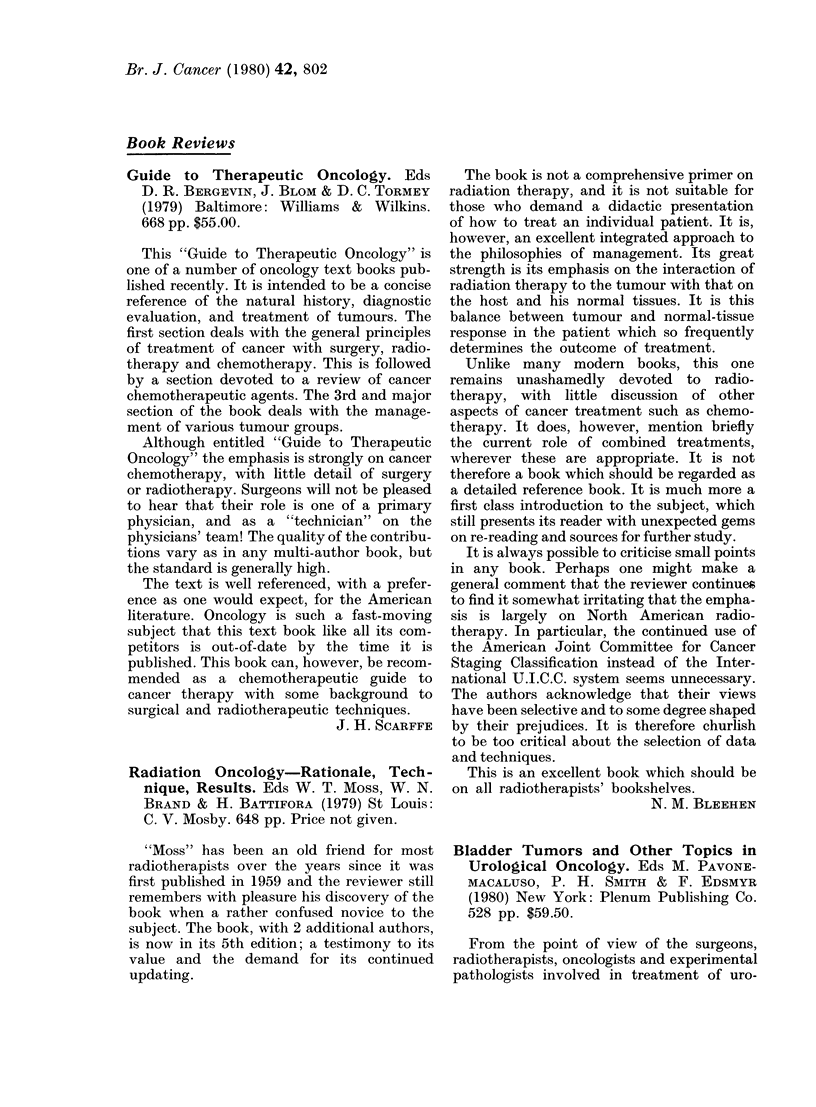# Guide to Therapeutic Oncology

**Published:** 1980-11

**Authors:** J. H. Scarffe


### *Book Reviews*

**Guide to Therapeutic Oncology.** Eds D. R. Bergevin, J. Blom & D. C. Tormey (1979) Baltimore: Williams & Wilkins. 668 pp. $55.00.

This "Guide to Therapeutic Oncology" is one of a number of oncology text books published recently. It is intended to be a concise reference of the natural history, diagnostic evaluation, and treatment of tumours. The first section deals with the general principles of treatment of cancer with surgery, radiotherapy and chemotherapy. This is followed by a section devoted to a review of cancer chemotherapeutic agents. The 3rd and major section of the book deals with the management of various tumour groups.

Although entitled "Guide to Therapeutic Oncology" the emphasis is strongly on cancer chemotherapy, with little detail of surgery or radiotherapy. Surgeons will not be pleased to hear that their role is one of a primary physician, and as a "technician" on the physicians' team! The quality of the contributions vary as in any multi-author book, but the standard is generally high.

The text is well referenced, with a preference as one would expect, for the American literature. Oncology is such a fast-moving subject that this text book like all its competitors is out-of-date by the time it is published. This book can, however, be recommended as a chemotherapeutic guide to cancer therapy with some background to surgical and radiotherapeutic techniques.

J. H. Scarffe